# Implications for the migraine SNP rs1835740 in a Swedish cluster headache population

**DOI:** 10.1186/s10194-018-0937-0

**Published:** 2018-11-01

**Authors:** Caroline Ran, Carmen Fourier, Margret Zinnegger, Anna Steinberg, Christina Sjöstrand, Elisabet Waldenlind, Andrea Carmine Belin

**Affiliations:** 1 Department of Neuroscience, Karolinska Institutet, Biomedicum D7, Solnavägen 9, 171 65 Stockholm, Sweden; 2 Department of Clinical Neuroscience, Karolinska University Hospital, Stockholm, Sweden

**Keywords:** MTDH, rs1835740, PRDM16, rs2651899, Neurovascular headache, Association

## Abstract

**Background:**

Cluster headache is a severe headache disorder with unknown aetiology. The pathophysiology and symptoms present certain common features with migraine. Specifically, activation of the trigeminal vascular system seems to be involved in both disorders, which is hypothesized to result in neurogenic inflammation and vasodilation of the cerebral vessels. In addition, genetic factors have been implicated in both migraine and cluster headache.

**Objective:**

In order to determine whether or not migraine and cluster headache share genetic risk factors, we screened two genetic variants known to increase the risk of migraine in Sweden in a Swedish cluster headache case-control study population.

**Methods:**

In all, 541 patients and 581 control subjects were genotyped for rs1835740 in close proximity to *MTDH* (metadherin) and rs2651899 in the *PRDM16* (PR/SET domain 16) gene, using TaqMan® real-time PCR and pyrosequencing. In addition, we analyzed *MTDH* gene expression in a subset of the material, using reverse transcription real-time PCR to determine relative mRNA levels in primary fibroblast cell lines from patients and controls.

**Results:**

We found a trend for association between rs1835740, which is reported to affect *MTDH* mRNA levels, and cluster headache in our Swedish case-control material (*p* = 0.043, Χ^2^ = 4.102). This association was stronger in a subgroup of patients suffering from both cluster headache and migraine (*p* = 0.031, Χ^2^ = 6.964). We could further confirm that rs1835740 has an effect on the transcriptional activity of *MTDH*. In this Swedish cluster headache cohort we did not find an association with the rs2651899 variant.

**Conclusions:**

We conclude that rs1835740 is a potential risk factor for cluster headache in Sweden. Our data indicates that rs1835740 and *MTDH* might be involved in neurovascular headaches in general whilst rs2651899 is specifically related to migraine.

## Background

Migraine and cluster headache (CH) are two primary headache disorders that share pathological features and a majority of CH patients are successfully treated with drugs used for migraine, such as triptans [1–4]. Both disorders are characterized by activation of the trigeminovascular system and neurogenic inflammation [5]. There are also phenotypic similarities between CH and migraine, such as recurring attacks of headache, lateralized pain and associated autonomic symptoms [3, 4]. However in migraine these symptoms are typically nausea and vomiting while in CH autonomic symptoms are e.g. rhinorrhea, lacrimation and ptosis. Sometimes tearing of the eye and nasal congestion at the site of pain as seen in CH can occur also in migraine. However, there are marked differences in the clinical manifestations and associated symptoms between migraine and CH. In particular, the diurnal and seasonal rhythmicity with which headache attacks can be observed in many CH patients is unparalleled in other primary headaches [6, 7].

Genetic factors are likely to influence the risk of developing both migraine and CH [8]. The heritability of CH is unclear, but having a first- or second-degree relative with CH greatly increases the risk of developing the disorder [9]. One study reports heritability (h^2^) for CH of 0.26, but this number is probably an underestimation since only 2.3% of the patients in the cohort reported a familial history [10]. In our Swedish CH material, 11.2% of the patients report that they have a first-, second- or third degree relative with CH [11]. Several candidate genes such as *HCRTR2* (hypocretin receptor 2), *ADH4* (alcohol dehydrogenase 4), *NOS* (nitric oxide synthase) and *CLOCK* (Circadian Locomotor Output Cycles Kaput) have been suggested to be associated with the disease, although results are conflicting [12–19]. In contrast the heritability of migraine has been estimated to be around 50% (h^2^ = 0.44) [8, 20]. The contribution of genetic factors to migraine has further been confirmed in several genome-wide association studies (GWAS) [21–25].

We have previously replicated the finding of an association for two of the established risk alleles for migraine in a Swedish migraine case-control population, rs2651899 in *PRDM16* (PR/SET domain 16) and rs1835740 located between *MTDH* (metadherin) and *PGCP* (carboxypeptidase Q) on chromosome 8q22.1 [26]. Association of the *PRDM16* gene to migraine has been confirmed in several replication studies and meta-analyses and it is now considered a well-established migraine candidate gene [27–32]. Replication studies on rs1835740 are less affirmative [33–36]. The *MTDH* gene was found to associate with migraine in a gene-based multimarker analysis, but the rs1835740 variant did not reach genome-wide significance in a large GWAS meta-analysis [24, 31].

There are indications of CH being more common in families with migraine [37]. Moreover there are genes that have been suggested as candidate genes for both disorders, for example the *MTHFR* (methylenetetrahydrofolate reductase), *HCRTR2* and *iNOS* (inducible NOS) genes, but there are no conclusive reports on shared genetic risk factors [13, 16, 38–43].

The aim of this study was to investigate whether migraine and CH might share genetic determinants since similar pathophysiological events occur in both disorders. Analysis of the distribution of the genetic migraine markers rs2651899 and rs1853740 has not previously been performed in CH. We therefore screened a large Swedish CH case-control population and performed an association analysis of these two markers that have been shown to increase the risk for migraine in Sweden.

## Material and methods

### Genetic analysis

The material consisted of 541 CH cases and 581 controls where 571 of the controls were anonymous healthy blood donors, these samples were obtained from local blood donation centres in 2003–2005. DNA was isolated from whole blood according to standard procedures after written informed consent. Participating research subjects were required to fill in a questionnaire assessing clinical and lifestyle parameters. Medical journals of all 541 CH patients were reviewed by a neurologist (one of the co-authors A.S., E.W. or C.S.) in order to confirm the diagnosis according to the International Classification of Headache Disorders (ICHD-II) [44]. 518 CH patients were from our carefully characterized Swedish CH biobank recruited during the years of 2013–2017 [11]. 23 of the CH cases were obtained from the TwinGene study, conducted between 2004 and 2008, the procedure of identifying individuals with cluster headache, blood sampling, genotyping and quality control of the array data has been described in previous publications [26, 45]. In total, we analyzed 581 control individuals (56.3% male) and 541 unrelated CH cases with typical CH characteristics; predominantly male (67.7%), mean age of onset was 31.5 years. 464 patients (85.8%) suffered from episodic CH and 50 patients (9.2%) suffered from chronic CH at the time of sample collection, while 27 patients had un uncertain clinical phenotype. 10.9% had familial history of CH defined as having at least one first- or second degree relative with CH (a total of 467 patients provided information on heredity) and 16.4% also suffered from migraine (a total of 451 patients provided information on migraine status). All experiments were approved by the Regional Ethical Review Board, Stockholm, Sweden.

Genotyping was done with quantitative real-time PCR (qPCR), using an ABI 7500 Fast system (Applied Biosystems, Foster City, CA, USA) and TaqMan® reagents for allelic discrimination. TaqMan® genotyping master mix and predesigned single nucleotide polymorphism (SNP) assays for the two SNPs (assay IDs C_11334245_10 (rs1835740) and C_27103073_10 (rs2651899)) were ordered from Applied Biosystems/Life Technologies (Life Technologies Europe BV, Stockholm, Sweden). 2-5 ng dried DNA was used for each reaction, water served as negative control. The cycler was programmed as follows: pre-PCR read 60 °C for 1 minute and enzyme activation at 95 °C for 10 min, 50 cycles of 95 °C for 15 s and 60 °C for 1 minute, and post-PCR read 60 °C for 1 minute. Allelic discrimination was determined using the 7500 software v2.3. The call rate was 98.9% for C_11334245_10 and 98.6% for C_27103073_10.

Distinction between rs1835740 genotypes with the TaqMan® assay C_11334245_10 needed to be confirmed with an additional technique due to weak signal. One randomly chosen 96-whole plate of samples including all three potential genotypes was therefore re-genotyped using pyrosequencing. Primers were designed using ApE v2.0.49 (http://biologylabs.utah.edu/jorgensen/wayned/ape/), verified with the mfold software v3.6 and NCBI online Blast tool (https://blast.ncbi.nlm.nih.gov/Blast.cgi), and ordered at Thermo Fisher Scientific (Thermo Fisher Scientific, Hägersten, Sweden) [46]. Forward primer (5’-GTTGGAAGTGGGTGTCAGACC-3′), biotinylated reverse primer (5’-GCAGACTTTGGACAGTTCAGAA-3′), and sequencing primer (5’-AACTTGATTCCAATC-3′). DNA fragments were amplified with a regular PCR. The amplified fragments were then fixed to streptavidin-sepharose beads using a PyroMark Vacuum Prep tool (Biotage AB, Uppsala, Sweden), washed and released into an annealing plate containing the pyrosequencing primer. This plate was incubated at 80 °C for 2 min to anneal the primer followed by processing in an automated pyrosequencing PSQ 96 System using PyroMark Gold reagents (QIAGEN Nordic, Sollentuna, Sweden). The results, which were analyzed with the software provided with the PSQ 96 System and manually reviewed, were found to correspond to those obtained with TaqMan® qPCR.

### Gene expression analysis

Primary fibroblast cell lines derived from 12 CH patients and 12 control subjects were obtained from skin biopsies performed on the inside of research subjects’ upper arm. The biopsies were obtained under local anaesthesia, after informed consent. All experiments were approved by the Regional Ethical Review Board, Stockholm, Sweden. The biopsy was dissected into several smaller pieces and immediately cultured under a coverslip according to a protocol described by Akira Takashima [47]. Once established, the fibroblast cell lines were passaged two times, frozen in fetal bovine serum (FBS) with 10% DMSO and kept at − 150 °C for long-term storage.

Cells from the 12 CH patients and 12 control subjects were thawed and cultured in 75-cm^2^ culture flasks (Corning Inc., Corning, NY, USA) using DMEM GlutaMAX™ medium (25 mM D-Glucose and 1 mM sodium pyruvate) supplemented with 15% FBS, 1% 10 mM HEPES and 1% Antibiotic-Antimycotic (10,000 U/mL penicillin, 10,000 mg/mL streptomycin, and 25 mg/mL Amphotericin B), (all from Invitrogen, Paisley, UK), in a humidified 37 °C, 5% CO_2_ incubator. Cells were passaged once, and then harvested according to a protocol described by Johansson et al. [48]. RNA was prepared directly from frozen cell pellets using an RNeasy Mini Kit (QIAGEN) according to the manufacturer’s instructions. Further, the QuantiTect Reverse Transcription Kit (QIAGEN) was used to prepare cDNA from a total of 2 μg RNA.

The reverse transcription qPCR (qRT-PCR) was performed using a standard qRT-PCR program with 45 cycles of denaturation at 95 °C for 15 s and annealing/extension at 60 °C for 1 minute. We used an ABI 7500 Fast instrument (Applied Biosystems) and a master mix consisting of Power SYBR® Green PCR Master Mix (Thermo Fisher Scientific), primers at a concentration of 0.25 μM and approximately 200 ng cDNA. Primer sequences for *MTDH* and for the commonly used reference gene Pyruvate Dehydrogenase E1 Beta (*PDHB)* were obtained from the literature and ordered from Thermo Fisher Scientific [49, 50]. Both primer pairs had similar R^2^ values and efficiency: *MTDH* 0.996/ 105.9% and *PDHB* 0.990/ 101.8%. One control sample was a suspected outlier and was removed from the analysis after verification with Grubb’s test (Z > 2.4, significance level 0.05). A sensitivity analysis including all samples was performed, which gave a slightly lower *p*-value and thereby not affecting the outcome of the analysis. *MTDH* mRNA levels were normalized to the expression of *PDHB* and further normalized to a reference sample consisting of pooled cDNA from all 24 individuals.

### Statistical analysis

Genotype and allele association was analyzed using a two-tailed chi-squared (Χ^2^) test in GraphPad Prism v5.04 (GraphPad Softwares Inc., La Jolla, CA, USA). Our study design also comprised a subgroup analysis, stratifying patients according to migraine status to evaluate if the occurrence of migraine might affect the results. We did not perform power calculations prior to the study since our sample size was limited by sample availability. A post hoc power calculation based on the available sample size and a minor allele frequency (MAF) of 0.2 indicated that we can detect an odds ratio (OR) < 0.637 or > 1.485 with 80% power, which is within the same order of magnitude as our results (OR_rs1835740_ = 1.25, 95% confidence interval (CI) 1.01–1.53). Both SNPs were tested for Hardy-Weinberg equilibrium (HWE) in both patients and controls using the online OEGE HWE calculator [51].

The 7500 software v2.3 (Applied Biosystems), GraphPad Prism v5.04 and GraphPad QuickCalcs online tools (GraphPad Softwares Inc) were used for gene expression data analysis. mRNA levels were compared using an unpaired two-tailed t-test. Grubb’s test was used to discern outliers.

## Results

We genotyped 541 CH patients and 581 controls for the rs2651899 and rs1835740 SNPs, and tested for association with CH; results are represented in Table 1. For rs1835740, we found a weak association between the rare allele (T) and an increased risk for CH (Chi-square (Χ^2^) =4.1, OR 1.25, 95% CI 1.01–1.53, *p*-value = 0.043). Both the TT and the CT genotypes were more common in patients than controls, but there was no significant genotypic association. There was no association between rs2651899 in *PRDM16* and CH in our material (Table 1). Both SNPs were in HWE in patients and controls (data not shown).

**Table 1:** Results from the genotype and allele analysis

SNP	Genotype/Allele	Controls % (n)	CH % (n)	χ^2^ (df)	OR (95% CI)	*p*-value
rs1835740	CC	66.6 (379)	61.7 (334)			
	CT	29.7 (169)	32.3 (175)			
	TT	3.7 (21)	5.9 (32)	4.524 (2)	–	0.1
	C	81.5 (927)	77.9 (843)			
	T	18.5 (211)	22.1 (239)	4.102 (1)	1.25 (1.01–1.53)	0.043
rs2651899	TT	33.2 (189)	37.1 (199)			
	TC	49.6 (283)	45.3 (243)			
	CC	17.2 (98)	17.5 (94)	2.340 (2)	–	0.31
	T	58.0 (661)	59.8 (641)			
	C	42.0 (479)	40.2 (431)	0.676 (1)	0.93 (0.78–1.1)	0.41

*SNP* single nucleotide polymorphism, *n* number, *CH* cluster headache, *χ*^2^ chi-squared, *df* degrees of freedom, *OR* odds ratio, * 95% CI* 95% confidence interval

Since these two SNPs are known to influence the risk of migraine, and many CH patients in our material also suffer from migraine, we performed a stratified analysis with respect to migraine. Patients were separated into two groups; one consisting of patients suffering from CH only, and one consisting of CH patients with migraine. The allelic association of rs1835740 became stronger when analyzing CH patients with migraine, CH patients without migraine, and controls (Χ^2^ = 6.96, *p*-value = 0.031) (Table 2). When comparing the allele and genotype frequencies, the mutated allele became increasingly more common in the group of patients suffering from both CH and migraine; 21.1% in CH patients and 26.2% in CH patients with migraine, as compared to the control group where the MAF was 18.5%. The stratified analysis did not affect the results for rs2651899.

**Table 2:** Results from the genotype and allele analysis in patients with and without migraine

SNP	Genotype/Allele	Controls % (n)	CH without migraine % (n)	CH with migraine % (n)	X^2^ (df)	*p*-value
rs1835740	CC	66.6 (379)	63.4 (279)	54.5 (55)		
	CT	29.7 (169)	30.9 (136)	38.6 (39)		
	TT	3.7 (21)	5.7 (25)	6.9 (7)	7.391 (4)	0.12
	C	81.5 (927)	78.9 (694)	73.8 (149)		
	T	18.5 (211)	21.1 (186)	26.2 (53)	6.964 (2)	0.031
rs2651899	TT	33.2 (189)	34.9 (152)	47.0 (47)		
	TC	49.7 (283)	47.5 (207)	36.0 (36)		
	CC	17.2 (98)	17.7 (77)	17.0 (17)	8.029 (4)	0.091
	T	58.0 (661)	58.6 (511)	65.0 (130)		
	C	42.0 (479)	41.4 (361)	35.0 (70)	3.50 (2)	0.17

*SNP* single nucleotide polymorphism, *n* number, *CH* cluster headache, *χ*^2^ chi-squared, *df* degrees of freedom

**Fig. 1:**
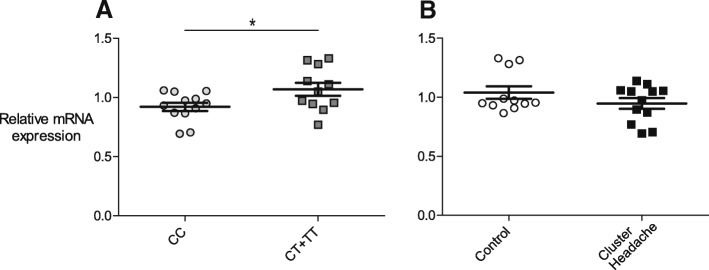
*MTDH* mRNA expression is affected by rs1835740. *MTDH* mRNA expression levels were normalized to the reference gene *PDHB* and to a reference sample consisting of pooled cDNA from all individuals. Horizontal bars represent group means, SEM in error bars, groups were compared using an unpaired, two-tailed t-test. **a**) mRNA levels are compared between individuals with two major alleles (CC) and individuals carrying one or more T alleles (CT + TT) at rs1835740 regardless of headache diagnosis, *p* = 0.0318. **b**) mRNA levels were compared between healthy control subjects and individuals with cluster headache, *p* = 0.195

The genetic association discovered here between CH and rs1835740, in combination with the discovery study reporting that rs1835740 potentially affects the expression levels of the *MTDH* gene lead us to also investigate the *MTDH* mRNA expression levels in a subset of patients and controls [21]. mRNA was obtained from primary fibroblast cell lines provided from 12 patients and 12 control individuals and analyzed using qRT-PCR. *MTDH* mRNA expression was normalized to *PDHB*, which was used as a reference gene, and then to a reference sample. One control individual was identified as an outlier using Grubb’s test and was removed from the analysis. We found a significant difference in the mRNA levels of *MTDH* when comparing individuals carrying two C alleles (wild-type) at rs1835740, with individuals having one or two T alleles, *p* = 0.0318, Fig. 1a. There was only one individual carrying the minor allele at both positions in our material, which is the reason we could not compare the three individual genotypes. We further analyzed the mRNA levels with respect to CH diagnosis, as can be observed in Fig. 1b, control subjects and CH patients had similar *MTDH* mRNA levels (*p* = 0.195).

## Discussion and conclusion

We have analyzed a Swedish CH population for two SNPs that have previously been identified as genetic risk factors for migraine in Sweden and globally [21, 22]. One of the variants, rs1835740, was found to be associated with an increased risk for CH. The other SNP, rs2651899, was not associated with CH in Sweden.

A more detailed analysis of rs1835740 revealed that the association was even stronger in a subgroup of patients that have both CH and migraine. When comparing the MAF between the three groups, we found that the minor allele (T) was present in 26.2% of the individuals suffering from both disorders, which is 7.7% more than among controls, a significant difference (*p* = 0.016) with an OR of 1.56 (95% CI 1.10–2.21), and 5.1% more than in CH without migraine. This difference was not significant possibly because of the smaller sample sizes in the stratified analysis. Our findings thus appear to indicate that the T allele is enriched in the patient group having both CH and migraine. In a former GWAS suggesting *MTDH* as a candidate gene for migraine, the effect of the rs1835740 association was stronger in a subgroup of patients suffering from migraine with aura (MA) than in patients with migraine without aura (MO), which is also an indication of this SNP being associated with more complex headache phenotypes [21]. Interestingly, in a study on rs1835740 and migraine by Azimova et al. (2015) small groups of CH patients (*n* = 9) and chronic tension type headache patients (*n* = 20) were used as control groups. In their study, all chronic tension headache patients were wild type for rs1835740, while the MAF of CH patients was 44.4% [36]. One possible limitation of our study is the lack of clinical information related to our control material. We cannot exclude the occurrence of headache in the control group; statistically, the occurrence of CH should be extremely low, or non-existent, but we have to assume that around 15% of our controls also suffer from migraine, corresponding to the prevalence of migraine in the Swedish population. In view of our data, the occurrence of migraine in the control group would imply a higher MAF of rs1835740, and should therefore not introduce a risk of false positive results.

*MTDH* has been suggested to be a likely candidate gene for migraine because of its proximity to the intergenic SNP rs1835740. Moreover, rs1835740 was found to affect the gene expression of *MTDH*, thus providing an appealing pathophysiological mechanism for migraine: *MTDH* is known to downregulate *EAAT2* (excitatory amino acid transporter 2), a major glutamate transporter, and could thus affect glutamate regulation at a synaptic level [52, 53]. We studied the *MTDH* mRNA levels in individuals with different rs1835740 genotype, and also analyzed expression with respect to CH diagnosis. Similarly to previous reports, the relative quantification showed a difference in expression between individuals with different genotypes for rs1835740 [21]. Individuals carrying one or two T alleles had significantly higher mRNA levels than individuals with the CC genotype. eQTLs (expression quantitative trait loci) may vary between different types of tissue and although gene expression in primary fibroblasts does not reveal how *MTDH* is expressed in nervous tissue, we were encouraged to be able to confirm the initial finding that associated the T allele of rs1835740 with increased *MTDH* expression in immortalized lymphocytes [21, 54]. There was no difference in mRNA levels between CH patients and controls.

The discovery of rs1835740 and *MTDH* provided a molecular link between migraine and familial hemiplegic migraine (FHM), a familial form of migraine caused by mutations in ion channels, since mutations associated with FHM subtypes one and two are known to cause increased levels of glutamate at the synaptic cleft [55, 56]. Similarly, our results suggest that *MTDH* and glutamatergic mechanisms may be involved also in CH pathophysiology. There are previous indications that glutamate might play a role in CH as well. A recent publication demonstrated a decrease in kynurenine metabolites, known glutamate receptor antagonists, in CH patients [57]. This could potentially affect the activity of the glutamate receptor NMDA and nociceptive sensitization [57, 58].

We conclude that rs1835740 in *MTDH* is associated not only with migraine but also with CH, whilst rs2651899 in *PRDM16* seems to be specifically related to migraine in Sweden. In view of our stratified analysis showing that the T allele of rs1835740 was more common in patients with both disorders, we also suggest that rs1835740 might constitute a genetic risk factor for more complex headache phenotypes, for example a combination of different primary headache syndromes as illustrated in our study. The T allele of rs1835740 was further shown to affect *MTDH* mRNA expression in primary fibroblasts. In depth analyses of *MTDH* and the rs1835740 SNP in CH and in other primary headache disorders would be of interest in order to determine to what extent rs1835740 is related to severe headache.

## Abbreviations

*ADH4*: Alcohol dehydrogenase 4
CH: Cluster headache
CI: Confidence interval
*CLOCK*: Circadian Locomotor Output Cycles Kaput
DF: Degrees of freedom
eQTL: Expression quantitative trait loci
FBS: Fetal bovine serum
FHM: Familial hemiplegic migraine
GWAS: Genome-wide association study
*HCRTR2*: Hypocretin receptor 2
HWE: Hardy-Weinberg equilibrium
ICHD: International Classification of Headache Disorders
*iNOS*: Inducible NOS
MA: Migraine with aura
MAF: Minor allele frequency
MO: Migraine without aura
*MTDH*: Metadherin
*MTHFR*: Methylenetetrahydrofolate reductase
*NOS*: Nitric oxide synthase
OR: Odds ratio
*PDHB*: Pyruvate Dehydrogenase E1 Beta
*PGCP*: Carboxypeptidase Q
*PRDM16*: PR/SET domain 16
qPCR: Quantitative real-time PCR
qRT-PCR: Reverse transcription qPCR
SNP: Single nucleotide polymorphism
χ^2^: Chi-squared
